# A formal evaluation of The Ottawa Hospital Pain Clinic orientation session: A quality improvement project

**DOI:** 10.1080/24740527.2022.2111993

**Published:** 2023-01-10

**Authors:** Patricia A. Poulin, Louise Bell, Danielle Rice, Yaadwinder Shergill, Sarah Fitzgerald, Rosemee Cantave, Renée Gauthier, Rose Robbins, Cristin Kargus, Susan Ward

**Affiliations:** a Department of Anesthesiology, The Ottawa Hospital Pain Clinic, Ottawa, Ontario, Canada; b Clinical Epidemiology, The Ottawa Hospital Research Institute, Ottawa, Ontario, Canada; c Department of Anesthesiology and Pain Medicine, Faculty of Medicine, University of Ottawa, Ottawa, Ontario, Canada; d Department of Psychology, Experimental Psychology, Memorial University of Newfoundland, St. John’s, Newfoundland, Canada; e Department of Anesthesiology, The Ottawa Hospital Research Institute, Ottawa, Ontario, Canada; f Department of Anesthesiology and Pain Medicine, The Ottawa Hospital Research Institute | Institut de recherche de l’Hôpital d’Ottawa, Ottawa, Ontario, Canada

**Keywords:** chronic pain, pain, qualitative research, quality assurance, pain education

## Abstract

**Background:**

Chronic pain affects approximately one in every five Canadians and has a substantial impact on psychological well-being, relationships, ability to attend work or school, and overall functioning.The Ottawa Hospital Pain Clinic introduced orientation sessions, with the aim of providing new patients with pain education to help prepare patients for engagement with multimodal pain management strategies. This report summarizes the results of a formative evaluation of the orientation session at The Ottawa Hospital Pain Clinic to determine whether patients perceived the orientation session as beneficial.

**Methods:**

Interviews were conducted, transcribed, and then thematically analyzed to understand patients’ perspectives on the orientation session. Coding was done by two team members using the constant comparison analyses method with key ideas, concepts, and patterns identified and compared to identify similarities.

**Results:**

Between September 6 and October 18, 2019, 18 patients attended an orientation session and 12 consented to participation and completed telephone interviews. The six themes identified included (1) feeling of community, (2) participants feeling heard by providers, (3) appreciation of the holistic approach, (4) availability of community resources, (5) barriers to access, and (6) discordant feelings of preparedness for the physician appointment.

**Conclusion:**

Results from this evaluation indicate that the orientation session offered at The Ottawa Hospital Pain Clinic improves chronic pain literacy, reduces feeling of isolation, and instills hope. As such, it appears to be a valuable component of pain clinic programs.

## RÉSUMÉ

**Contexte:** La douleur chronique touche environ un Canadien sur cinq et a des répercussions sur le bien-être psychologique, les relations, la capacité à aller au travail ou à l'école, et l'ensemble du fonctionnement. La Clinique de la douleur de l'Hôpital d'Ottawa a lancé des séances d'orientation, dans le but de fournir aux nouveaux patients une formation sur la douleur pour les aider à se préparer à adopter des stratégies multimodales de prise en charge de la douleur. Ce rapport résume les résultats d'une évaluation formative de la séance d'orientation à la Clinique de la douleur de l'Hôpital d'Ottawa visant à déterminer si les patients perçoivent la session d'orientation comme bénéfique.

**Méthodes:** Les entrevues ont été menées, transcrites, puis analysées de maniére thématique pour comprendre les points de vue des patients sur la séance d'orientation. Le codage a été effectué par deux membres de l'équipe à l'aide d'une méthode d'analyse par comparaison constante avec des idées, des concepts et des modéles clés répertoriés et par rapport à l'identification de similitudes.

**Résultats**: Entre le 6 septembre et le 18 octobre 2019, 18 patients ont assisté à une séance d'orientation. Parmi ceux-ci, 12 ont accepté de participer et ont complété des entrevues téléphoniques. Les six thémes répertoriés comprenaient (1) le sentiment de communauté, (2) le sentiment des participants d'étre entendus des prestataires, (3) l'appréciation de l'approche holistique, (4) la disponibilité des ressources communautaires, (5) les obstacles à l'accés, et (6) des sentiments discordants de préparation pour le rendez-vous chez le médecin.

**Conclusion**: Les résultats de cette évaluation indiquent que la séance d'orientation offerte à la Clinique de la douleur de l'Hôpital d'Ottawa améliore la littératie en matiére de douleur chronique, réduit le sentiment d'isolement et suscite l'espoir. Ainsi, elle semble étre un élément précieux des programmes de la Clinique de la douleur.

## Introduction

Chronic pain is defined as an aversive sensory and emotional experience typically caused by actual or potential tissue injury.^1^ Chronic pain may be a symptom of an underlying condition (chronic secondary pain) or it may be a person’s chief complaint, where pain and its associated distress and disability are not adequately explained by an underlying condition.^2^ Chronic pain affects approximately one in every five Canadians^3^ and has a substantial impact on psychological well-being, relationships, ability to attend work or school, and overall functioning.^4,5^ As such, chronic pain management usually requires a biopsychosocial approach taking into account the complex interaction between physical, psychological, and social factors that influence the experience of pain and concomitant disability.^6^

Multidisciplinary pain clinics generally use a biopsychosocial framework^6^ to inform treatments. However, patients referred to such clinics may have different expectations about pain treatments. This has been frequently observed in our and other specialty pain clinics, where patients often expect or hope for a medical cure.^7–9^ It appears that the biggest discrepancy between patient expectations for pain support and pain support delivered is the extent to which those supports can relieve patients’ pain. For example, in a pre-intervention survey, Sanderson et al. found that a reduction in pain (e.g., >50.9% reduction pre- versus post-care) was rated by patients (*n* = 24) as the most desirable and valuable outcome of the pain intervention offered at the clinic. However, in that same sample, when the interventions included brief cognitive behavioral therapy and/or opioid medication, the average reduction of pain was merely 11.9%.^9^ Access to comprehensive biopsychosocial programs in specialty pain clinics, such as education and interventions on the impact of poor sleep on pain catastrophizing, depression, anxiety, and quality of life, is vital to meeting patient expectations.^7^ Participation in multidimensional management programs may positively impact pain outcomes.

In order to address patient expectations and to help prepare patients for engagement with multimodal pain management strategies, The Ottawa Hospital Pain Clinic developed an orientation session that was, at the time this article was written, offered in person to all new patients. Following COVID-19-related disruptions in care, the orientation session is offered virtually and covers the same material. The orientation session takes place prior to the patient’s appointment with a specialist at the pain clinic and represents a window of opportunity intervention that aims to achieve multiple objectives, including (1) providing pain science education, (2) encouraging patients to develop realistic expectations about the pain clinic, (3) orienting them to pain clinic processes and procedures, (4) improving readiness to engage with the various programs offered by the pain clinic, (5) increasing awareness of community resources, and (6) instilling hope.

The cornerstone of the orientation session is pain education, which aims to provide information about the psychological, physical, and social processes of chronic pain.^10^ This may reduce the fear of pain through understanding that pain does not necessarily mean harm (hurt versus harm).^11^ Another aim of pain education is to begin exploring pain management strategies that may be helpful to reduce the impact of pain (e.g., pacing of activities, improving sleep). Resources available for patients to start addressing co-occurring problems are also provided (e.g., drop-in counseling and other community resources). This is based on the premise that improving knowledge of pain and providing concrete strategies for patients to explore while waiting for their clinic appointment will be associated with better self-management and clinical outcomes.^12,13^

Given that the orientation session is the first formal point of contact between patients and the clinic, it is important to understand patients’ experiences of participating in this session. This article summarizes the results of a formative evaluation of the orientation session within our tertiary pain center. A formative evaluation is an iterative assessment process that detects influences on the progress and effectiveness of implementing a new intervention, program, or strategy.^14^ Formative evaluations begin early in an implementation process and the results are shared with the study team. This approach is particularly helpful when conducting continuous quality improvements because it allows for timely, transparent, and separate evaluations of the intervention and implementation processes. Changes can be made, and outcomes can be monitored at any stage of the process.

A formative evaluation approach was used to determine whether patients perceive the orientation session as beneficial, whether they are satisfied with the session, and whether the session leads to increased readiness to engage with programs at the clinic. We also aimed to understand the strengths, gaps, and recommendations for improvement as perceived by patients that would help improve the session and overall patient experience.

## Methods

### Design

The overall framework for the development and evaluation of our orientation session is the continuous quality improvement cycle (plan, do, study, act).^15^ To understand participants’ experiences of the session and their recommendations for improvement, we conducted one-on-one telephone interviews and thematically analyzed the interview data. The protocol for the evaluation was reviewed by The Ottawa Health Sciences Network Research Ethics Board and received exemption from internal review due to the project aligning with quality improvement goals. The protocol was registered with The Ottawa Hospital Quality Improvement Department and this report is informed (by)^16^ the Revised Standards for Quality Improvement Reporting Excellence (SQUIRE 2.0).^17^

### Setting

This evaluation was conducted through The Ottawa Hospital Pain Clinic, located in an urban tertiary academic medical center in Ontario, Canada. Data were collected from September 17 to October 22, 2019. At the time of this evaluation, The Ottawa Hospital Pain Clinic’s core interprofessional team was funded by the Ontario Ministry of Health. The team responsible for the development of the pain clinic orientation session included two psychologists, one social worker, one physiotherapist, and one occupational therapist. Patients eligible to receive treatment at The Ottawa Hospital Pain Clinic must have chronic primary or secondary pain and need to have a family physician.

The clinic offers its services both through direct care as well as through asynchronous specialist consultation (Champlain BASE, eConsult).^18^ The orientation session is offered to patients in our direct care stream. The pain clinic uses an interprofessional stepped care approach.^19^ In addition to medical management (e.g., procedures such as nerve blocks, radiofrequency ablation, intravenous infusions, medication review), patients are offered a suite of programs that they can integrate within their individualized care plan, including psychoeducational workshops on various topics (e.g., sleep, pacing, coping with grief and loss, exercising with chronic pain), a support group (e.g., social work–led weekly support group), and therapy groups (e.g., mindfulness-based pain management, cognitive-behavioral therapy for anxiety and depression, dialectical behavior therapy skills group).

Furthermore, an 8-week chronic pain management program, drop-in mindfulness and exercise for chronic pain sessions, and individual consultation and treatment with members of the interprofessional team are offered to patients of the pain clinic. Treatment is individualized, meaning that patients can generally expect to receive pain clinic care for up to 1 year; however, some patients will have a single consultation, whereas others will receive treatment for many years. There was no wait to access the pain clinic orientation session at the time this evaluation took place. There was, however, an estimated 6- to 12-month wait time for a physician visit depending on the reason for referral and no wait to access interprofessional team programs following the physician visit.

### Orientation Session

The Ottawa Hospital Pain Clinic interprofessional team developed and implemented an orientation session as part of the intake process for all new patients. The content of the orientation session focuses on pain science, best practices in chronic pain management, community resources to help with managing pain (e.g., Ontario self-management programs), information about living healthy with various types of chronic pain, and information about our online portal for patients to view their hospital charts. The session is facilitated by a clinical psychologist and a social worker, lasts 90 min, and, at the time of the evaluation, was offered weekly, in person, to groups of up to ten patients per session due to space limitations precluding larger groups.

### Participants

All patients accepted into the pain clinic are encouraged to attend an orientation session prior to meeting with their assigned physicians. Exclusion criteria for the orientation session are (1) people who do not speak English, (2) individuals who are hard of hearing or deaf, (3) individuals with cognitive deficits or severe mental health conditions that precluded them from participating in a group session, and (4) patients who recently transitioned from pediatric care or emergency care. Those meeting exclusion criteria are provided with similar information through alternative methods (i.e., their one-to-one consultation with nurses, physician, or members of the interprofessional team). No additional exclusion criteria were applied to the evaluation of the orientation session.

### Recruitment

Participants who attended the orientation session at the pain clinic from September 6 to October 18, 2019, were invited to participate in a 20-min telephone interview regarding their experience of the orientation session. Contact cards were provided to participants, who could then indicate their interest in participating in the interview. Those who wished to participate were asked to provide their phone number or e-mail and the time frame that best suited their availability and provided written consent to be contacted for the interview. Participants who preferred telephone were contacted at their requested time and those who provided an e-mail were e-mailed to schedule a telephone interview.

### Interview Guide and Development

The evaluation team (L.B., P.P.) developed an interview script designed to explore participants’ attitudes, experiences, perceptions, and satisfaction after attending the orientation session (see online supplement). The interview script was also reviewed by the health care providers (P.P., S.W.) who provide the orientation session for modifications prior to being implemented. Two interview guides were developed. One guide was developed to ask participants about their experience of the orientation session before attending their first physician appointment. The second guide was created to ask specific questions to individuals who had attended their first physician appointment after the orientation session but before the interview. Some questions in the second guide aimed to capture individuals’ opinions and experiences, such as whether they felt that the orientation session prepared them for their physician appointment.

Semistructured, open-ended interviews were completed over the telephone by a research assistant (L.B.) within the interdisciplinary pain research lab affiliated with the pain clinic. The research assistant had 2 years of experience and was a graduate student in psychology with experience in conducting patient interviews. The interviewer, along with the team, was motivated by a desire to understand and, if possible, assist in improving participant experience of the orientation session. The interviewer did not have any perceived conflicts of interest, because she was on a time-limited external fellowship from her master’s program and there were no previous relationships with any participants.

### Data Analysis

Interviews were analyzed using thematic analysis to explore patients’ perspectives on the orientation session. This process involves identifying and naming patterns of meaning that emerge from interview transcripts that relate to the research question.^20^ All interviews were audio-recorded and transcribed by a research assistant. Coding was done using the constant comparison analyses method^21^ in duplicate and independently by two members of the evaluation team with experience in chronic pain until saturation was reached (L.B., R.C.). One team member who analyzed results was the external master’s student, and the second team member held a research and administrative role in the pain clinic. Neither team member had relationships with the participants.

Key ideas, concepts, and patterns were recognized and compared to identify similarities. The resulting themes and supporting quotes were presented to the interprofessional team for interpretation and discussion of the implications of the findings. This approach to analysis is in line with recommendations from quality standards for qualitative research, whereby credibility is increased when there are multiple investigators analyzing data.^22^ This is also in line with the objectives of this formative evaluation to provide feedback to the team. De-identified data were imported into an Excel sheet, and all themes were imported into a Word document and are presented with representative quotes.

## Results

### Demographics

Between September 6 and October 18, 2019, 18 patients attended an orientation session and 12 consented to participation and completed telephone interviews. Reasons for nonparticipation included not responding to e-mail (*n* = 3), not responding to phone calls (*n* = 3), and difficulty with the English language (*n* = 1). Eight were female (66.7%) and 4 (33.3%) were male. The average age was 61 (SD = 14) years and the range of participant ages was 38–84.

Overall, six themes were identified from the interview transcripts. Themes were categorized as perceived strengths (themes 1–4) and weaknesses (themes 5–6) of the session. The themes included (1) feeling of community, (2) participants felt heard by providers, (3) appreciating the holistic approach, (4) availability of community resources, (5) barriers to access, and (6) discordant feelings of preparedness for the physician appointment.

### Strengths of Orientation Session

#### Theme 1: Feeling of Community

After attending orientation, participants felt a sense of community. They described leaving orientation with the feeling that they were not alone in experiencing their pain, feeling understood by others, and feeling a sense of unity with the individuals that were in their orientation group. Participants appreciated that other people conveyed similar experiences and struggles that they do:

P1: … having that group there and having somebody else ask that question really made me feel that I’m not alone in this and that when my pain does get lower on some days, I don’t really need to tell myself I’m crazy. … So, it’s nice to know that there are other people out there that are dealing with this and they actually know what I’m going through.

P11: … help and support from other people that are going through … ah, emotional support that helps you in different ways … you’re [not] fighting by yourself … you’re not alone.

#### Theme 2: Participants Felt Heard by Providers

After listening to the orientation session and hearing the variety of programs available at the clinic, participants expressed feeling hopeful. They appreciated that the clinic recognized that their pain exists and that there may be treatment options available to them through the clinic. Participants voiced that they finally felt as though their pain was being taken seriously:

P9: They understand pain … these real pain clinics that people can go to that people can talk to and go to to get help from.

P1: Hearing we believe you and we’re here to help you; that was a huge help.

#### Theme 3: Patients Appreciated the Holistic Approach

Participants described appreciating the variety of programs available at the clinic targeting medical, social, and psychological needs. They were pleased that the pain clinic was holistic in its approach, with a variety of resources available for people with different needs. In addition, appreciation was noted for the individualized nature of the pain management plan that could be developed based on patient’s needs, values, and preferences:

P10: It [pain clinic] touches on so many different things like whether relaxation techniques or um, the medications, um, physiotherapy, you know, just the whole variety of different things you look at and it’s not just one thing … this, it covers—it covers everything. And it covers a change in behavior, a change in thought, which is part of my— part of my problem, too, you know.

P11: Just that there are different options available. … Uh, other than just medication … that there are programs and there are relief up there for people for different needs. Who need emotional support, there’s programs for that. There are programs for running, for exercise, and whatnot for people who might need the extra healthy living, who might get the pain under control. So that was definitely brought to the forefront … it’s nice to have the extra resources.

#### Theme 4: Availability of Community Resources

Participants were unaware of the various resources that were available to them in the community. They appreciated gaining an awareness of the resources available and how to access them:

P4: I didn’t realize how many services were available in the Ottawa area. So that—that was good information for me particularly. …

P10: Different things available in the community that were, uh, basically available at no cost. … And the fact that you could put in for RDSP and the—the government would match them. I did not know that existed.

### Weaknesses of Orientation Session

#### Theme 5: Barriers to Access

Some participants identified that the main barrier to the pain clinic and the orientation session was the geographical barrier. Participants identified that they lived too far away from the clinic, so they were unable to attend the programs available after the orientation session:

P7: I’m from [outside of Ottawa]. … Unfortunately, it [the pain clinic] doesn’t really help me out a whole lot. … I’m very happy that these services are available for me, but unfortunately, I’m working full time and not able to participate in the pain clinic workshops.

P6: Well, I live in [outside of Ottawa] so, that’s, uh, we’re about a hundred kilometers away, so. …

Other participants identified that they are unable to take time off work to attend the Pain Clinic programs:

P3: I can’t take advantage of all the things that are offered because I just don’t have the time or sick days to keep taking time off work to do it.

Some participants appreciated that the orientation session at the pain clinic is available to them but decided to seek out resources that were available in their area instead of traveling to the clinic:

P7: I am thankful that there is a place in Ottawa for people who do suffer and have the resources available. …

#### Theme 6: Discordant Feelings of Preparedness for the Physician Appointment

After attending both the orientation and the physician appointment, there were mixed opinions regarding whether the orientation prepared them for their physician appointment.

Some people thought orientation prepared them well for their appointment:

P7: Um, it explained what the pain clinic is really all about and what the expectations are. How did it help me with my—well, I guess with the expectation that—that this appointment would not be like a quick fix or anything, that it’s just, um, providing strategies on how to help deal with pain management.

Other participants felt that attending the orientation did not prepare them enough for their physician appointment:

P12: They talked about coming to the next appointment, the appointment with the doctors, but they didn’t really describe the kind of interventions that might take place. … Just a little more explanation as to what they might do and why you would need somebody there with you.

Some participants gave suggestions as to what they would like to see at orientation regarding the physician appointment.

P9: … so, uh, I would like to see in the orientation that in the hour and a half that your scheduled for your—to see the pain doctor, be prepared that you’re going to be spending an hour going over all your medical history in—in detail and, um, spending very little time actually talking with the actual doctor.

P12: They could tell you just in a general way what kind of things they might be looking at to do and why you should have a car or driver waiting for you. … That part I found was a lapse. That they really didn’t fill you in, and that’s the most important part of the first stage … what sorts of things might happen. What sorts of things they might do and why you would need somebody with a car. … It was ambiguous.

In addition to uncertainty regarding the physician appointment, some participants identified that they were unprepared and uncertain of what was to be expected for medical procedures or interdisciplinary programs at the clinic. Some participants would have liked more detailed guidance on these topics before leaving orientation.

Some participants wanted more information regarding availability of different types of medical procedures:

P5: Um, well, it didn’t really cover what I thought I was going for. … I thought that I would be told a little bit more about the injections. … I was really anxious to hear about—more about the injections.

P6: We never spoke procedures at the orientation. Um, there was no information on what ketamine infusions were or nerve blocks or what type of evaluation we should expect from the doctor when we first show up. …

P3: I would’ve liked to have heard about medial branch ablations and—and—and you know some of the procedures they can do to actually um innervate certain areas so that decreases pain or gets rid of the pain all together.

Some participants wanted further instruction on interdisciplinary programs that are available after attending orientation.

P11: Like it would be nice, um, just giving an explanation of the overall clinic and how things are going to work, like first you see orientation then second you see your doctor. … I wish she maybe would have touched on the programs, the after clinic, the programs that you offer, only from the doctors, like the workshops and things that you have … but really it wasn’t fully given to us as far as what’s available, how do we access it, and how to get involved with it because, ah, they just spoke about it real quickly but nothing was given out, like no handouts.

P10: The only thing I would’ve liked more information on was just, um, I guess sort of like a pathway, like some people—I would be there every day for probably a couple of weeks, and some would be there for every—would it be once a week sort of thing? Like I just didn’t know how it worked. …

## Discussion

The current evaluation aimed to identify the strengths and gaps of the orientation session provided by The Ottawa Pain Clinic as perceived by patients who attended orientation. Several themes were identified as strengths of the orientation in addition to areas for improvement. Members of the interprofessional team collaborated to discuss themes and implications of findings.

Patients reported a sense of community within their groups and appreciated that many individuals were facing struggles similar to their own, resulting in a reduced feeling of isolation. Although not therapy, it appears that the experience of the orientation session aligns with the experience of group therapy, which provides social support for individuals and sentiment of common humanity.^23^ Group therapy can often result in feelings of encouragement and support of their peers while simultaneously reducing feelings of isolation.^23^ It also teaches individuals that they are not alone in experiencing their respective behavioral and psychological problems.^24^ Similar findings are in line with this notion, with peer support being perceived as the most important and valuable part of the chronic pain management program.^25^

Many participants also appreciated the empathetic approach provided by the facilitators. They felt that they were finally being heard, that their pain was acknowledged and validated, and that something was going to be done to help manage their pain. Empathy has been shown to improve patient perspectives on pain and health outcomes.^26^ Similar to our findings, Sternke and colleagues^27^ reported that patients who experience chronic pain feel acknowledged when their pain is believed by the provider. Participants appreciated knowing that they could voice their concerns and these concerns would be addressed. Providing empathy to patients can also improve better health outcomes and higher quality of treatment.^26,28^

Patients also appreciated the holistic approach provided by the pain clinic, which includes the use of medication while addressing the psychological, functional, and social aspects of pain. The biopsychosocial approach in multidisciplinary settings is considered best practice for the treatment and management of chronic pain.^29^ This is aimed at addressing the physiological, cognitive, social, and emotional processes that contribute to the experience of chronic pain.^30^ Programs are available at the pain clinic that are facilitated by medical professionals including but not limited to psychologists, a social worker, a physiotherapist, and an occupational therapist. It is evident as mentioned from participants in the orientation program that the opportunity to engage in diverse programs are appreciated.

The community resources available for participants in surrounding areas provided patients with a sense of autonomy and empowerment. Though some participants did not feel it necessary to use some of the resources at present, they appreciated that they could avail themselves of these resources if needed. As such, providing patients with a list of community resources can foster a sense of autonomy, because patients can utilize the resources when they feel it is appropriate. These resources were also important in the case where participants lived far away from the clinic and preferred not to or were unable to travel to attend pain clinic programs beyond their medical appointment.

Patients felt that access to the pain clinic was difficult for various reasons, including proximity or personal work requirements. Online delivery of orientation and programs within the pain clinic can help to remove the barrier of access due to geographical barriers.^31^ This has now been adopted for all interprofessional programs at the pain clinic because the COVID-19 pandemic limited access for in-person appointments.^32–34^ Other identified barriers that contribute to the ability to actively participate in programs at the pain clinic include language, hearing difficulties, or significant mental health impairment. These barriers highlight the importance of gathering the necessary information from the referring physician in order to provide appropriate accommodations for patients. For example, the orientation session could be recorded and dubbed in multiple languages; participants scheduled to attend an orientation session could be sent the presentation in their primary language and be accompanied by an interpreter for the question-and-answer period.

Patient expectations varied after the orientation session. Some participants hoped for more information about medical procedures available at the clinic. Other participants wanted more information about the interdisciplinary programs and workshops that were offered, and others hoped for an outline or plan on what to expect in the near future from the pain clinic. Because the pain clinic provides individualized care, addressing expectations in a group setting can be problematic. After discussion from the interprofessional team, plans for future orientation programs are to provide mock vignettes demonstrating different pain management plans. These vignettes will depict different pathways that will accurately portray how each individual plan can be different. The team additionally decided to include more information about allied health professional services available such as workshops and groups that are provided by the team.

There are some limitations to the study. Primarily, we sought to understand the strengths and gaps of the orientation session provided by the pain clinic at The Ottawa Hospital, which limits the transferability of our findings. Second, given that participants self-selected to partake in the interview, our findings may be prone to selection bias. Third, interviews took place by phone, which does not allow for interpretation of nonverbal information or allow for additional questions to be asked based on this information.

The adoption of an orientation session may be a beneficial component of multidisciplinary chronic pain programs. Overall, the orientation session appears to be achieving the goal of improving participants’ knowledge of chronic pain and its management and instilling hope; participants felt less alone as a result of their participation, felt heard, and trusted that the team would address their concerns. Participants provided useful feedback with regard to the need for more information about the medical interventions offered at the clinic as well as various programs offered and highlighted a need to address geographical, time, and language barriers to access. With the widespread adoption of virtual care during the COVID-19 pandemic, our team’s programs, including the orientation session, are now available virtually, facilitating access to care for many patients. Future evaluation will focus on the impact of the virtual delivery of the orientation session, along with the effect of this session on chronic pain knowledge and pain-related beliefs.

## Conclusion

Results from this evaluation indicate that the orientation session offered at The Ottawa Hospital Pain Clinic improves chronic pain literacy, reduces feeling of isolation, and instills hope. As such, it appears to be a valuable low-cost intervention to be integrated in pain clinic programs. There are alternative ways to deliver such information (e.g., text, video, self-directed courses), and future study could compare different strategies and modes of delivery along with their appropriate tailoring, to improve pain literacy, reduce isolation, increase readiness for engagement with pain self-management, and instill hope for people living with pain.

## Supplementary Material

Supplemental Material
