# Supplementary material for: A formal evaluation of The Ottawa Hospital Pain Clinic orientation session: A quality improvement project
Source: Can J Pain. 2023 Jan 10;7(1):2111993. doi: 10.1080/24740527.2022.2111993 (PMC9839373; doi:10.1080/24740527.2022.2111993)
Supplement: Supplemental Material [file UCJP_A_2111993_SM1705.docx]

**Appendix 1:**

**Semi-Structured Interview Questions for orientation program evaluation.**

Good morning/afternoon [insert participant name],

Thank you for setting aside the time for this interview. My name is [insert research assistant name] and I am a research assistant working with Dr. Patricia Poulin and I will be conducting your interview today. I wanted to take a moment to discuss the agenda for the interview. The interview will last between 15-20 minutes. I will ask you questions about your experience during the orientation you attended at the Pain Clinic. Please note that your candid responses are welcomed and needed to improve our orientation. Your responses will remain anonymous. After the interviews are transcribed, we will analyze the data focusing on themes as opposed to individual responses. We will use some quotes to illustrate these themes but these will be generic; no quotes that could permit identification will be used. Do you have any questions about that?

For quality improvement purposes, I would like to record our conversation. Is that okay with you?

[TURN ON RECORDER—placed so that you can see the little red light. Also, see notes from the transcribing service on how best to record a telephone interview.

With the recorder on, state your name, respondent’s name, and today’s date. Confirm that respondent has agreed to participate in the interview and to be recorded. Wait for audible affirmation.]

Do you have any questions before we get started? [*Respond to questions*].

1. After attending orientation, how prepared do you feel now about attending the programs at the pain clinic?
   a) what information made you feel more prepared about attending the program?
2. What information from the orientation stood out to you?
   a) Was this information helpful or unhelpful?
   b) What is the most important thing you remembered?
3. Was there any information provided during orientation that you think could be omitted?
4. What do you feel is the main take home message from orientation?
5. How did you like the interactive experience?
6. Do you feel there should be more, less, or no change?
   (remember when the facilitators asked questions for you to answer and tried to engage you at orientation, how did you like this?)
7. In your MyChart, there are governmental resources and community programs available. Have you had the chance to access this?
   1. If so, what resources did you access?
   2. If no, why haven’t you accessed this?
8. Is there a topic you wish was brought up during orientation but was not?
9. What did you expect to receive from orientation?
   1. Did the orientation meet your expectations?
      1. (if yes) How so?
      2. (if no) Why do you think that was?
10. Do you have suggestions to improve orientation?
    OR is there anything you would change to orientation?

Thank you for your time and your valuable input.

Do you have any questions for me?

**Semi-Structured Interview Questions for orientation program evaluation.**

Good morning/afternoon [insert participant name],

Thank you for setting aside the time for this interview. My name is [insert research assistant name] and I am a research assistant working with Dr. Patricia Poulin and I will be conducting your interview today. I wanted to take a moment to discuss the agenda for the interview. The interview will last between 15-20 minutes. I will ask you questions about your experience during the orientation you attended at the Pain Clinic. Please note that your candid responses are welcomed and needed to improve our orientation. Your responses will remain anonymous. After the interviews are transcribed, we will analyze the data focusing on themes as opposed to individual responses. We will use some quotes to illustrate these themes but these will be generic; no quotes that could permit identification will be used. Do you have any questions about that?

For quality improvement purposes, I would like to record our conversation. Is that okay with you?

[TURN ON RECORDER—placed so that you can see the little red light. Also, see notes from the transcribing service on how best to record a telephone interview.

With the recorder on, state your name, respondent’s name, and today’s date. Confirm that respondent has agreed to participate in the interview and to be recorded. Wait for audible affirmation.]

Do you have any questions before we get started? [*Respond to questions*].

1. How did the orientation prepare you for your physician appointment?
2. Did the physician recommend any programs at the pain clinic for you?
   1. If yes, see 3
   2. If no, skip 3
3. After attending both the orientation and physician visit, how prepared do you feel now about attending the programs at the pain clinic?
   a) What information made you feel more prepared about attending the program?
4. What information from the orientation stood out to you?
   a) Was this information helpful or unhelpful?
   b) What is the most important thing you remembered?
5. Was there any information provided during orientation that you think could be omitted?
6. What do you feel is the main take home message from orientation?
7. How did you like the interactive experience?
8. Do you feel there should be more, less, or no change?
   (remember when the facilitators asked questions for you to answer and tried to engage you at orientation, how did you like this?)
9. In your MyChart, there are governmental resources and community programs available. Have you had the chance to access this?
   1. If so, what resources did you access?
   2. If no, why haven’t you accessed this?
10. Is there a topic you wish was brought up during orientation but was not?
11. What did you expect to receive from orientation?
    1. Did the orientation meet your expectations?
       1. (if yes) How so?
       2. (if no) Why do you think that was?
12. Do you have suggestions to improve orientation?
    OR is there anything you would change to orientation?

Thank you for your time and your valuable input.

Do you have any questions for me?
